# Impact of General Anesthesia on Ciliary Functional Analysis by Digital High-Speed Videomicroscopy in Suspected Primary Ciliary Dyskinesia

**DOI:** 10.3390/diagnostics14212436

**Published:** 2024-10-31

**Authors:** Lionel Benchimol, Noemie Bricmont, Romane Bonhiver, Grégory Hans, Céline Kempeneers, Philippe Lefebvre, Anne-Lise Poirrier

**Affiliations:** 1 Department of ENT, University Hospital Liège, Avenue de l’Hôpital1, 4000 Liège, Belgium; pp.lefebvre@uliege.be; 2 Pneumology Laboratory, I3 Group, GIGA Research Center, University of Liège, 4000 Liège, Belgium; noemie.bricmont@chuliege.be (N.B.); rbonhiver@uliege.be (R.B.); ckempeneers@chuliege.be (C.K.); 3 Division of Respirology, Department of Pediatrics, University Hospital Liège, 4000 Liège, Belgium; 4 Department of Anaesthesia and Intensive Care Medicine, CHU of Liege, 4000 Liège, Belgium; g.hans@chu.ulg.ac.be

**Keywords:** primary ciliary dyskinesia, digital high-speed videomicroscopy, general anesthesia, ENT surgeries

## Abstract

Digital high-speed videomicroscopy (DHSV) is a crucial tool for evaluating ciliary function in children suspected of primary ciliary dyskinesia (PCD). However, until now, samples are taken without anesthesia due to uncertainty about its effect on ciliary function and DHSV interpretation. This study aimed to investigate the impact of general anesthesia on ciliary functional analysis by DHSV in a series of three patients listed for ENT surgeries, which could improve diagnostic procedures for pediatric patients. Patient 1 (7-year-old girl) underwent adenotonsillectomy and tympanostomy placement tube, while patients 2 (17-month-old boy) and 3 (15-month-old girl) underwent adenoidectomy and tympanostomy placement tube. All patients underwent nasal brushing before general anesthesia (control sample). Experimental samples were taken in the contralateral nostril at the time of equilibration of the anesthetic agents (sevoflurane, propofol, sufentanil). Ciliary beat frequency and pattern were measured using digital high-speed videomicroscopy. Our findings highlighted the variability of respiratory ciliary function under general anesthesia among individuals. Our results emphasize the need for caution when interpreting ciliary function data obtained during general anesthesia. Further research with larger cohorts is warranted for validation.

## 1. Introduction

Maintaining effective airway clearance and defense mechanisms relies on the production, drainage, and regulation of periciliary fluid and mucus, alongside the coordinated function of the ciliated epithelium. Impaired mucociliary clearance can stem from primary conditions such as cystic fibrosis or primary ciliary dyskinesia, or it may develop as a secondary consequence of toxic exposures, infections, or chronic diseases such as chronic rhinosinusitis [1,2].

Primary ciliary dyskinesia (PCD) is a rare genetic disorder characterized by disrupted ciliary function, leading to recurrent ENT and respiratory infections [2]. Its prevalence is difficult to determine, estimated between 1 in 10,000 and 1 in 20,000 people. However, the true prevalence of PCD is likely higher due to the complexity of diagnosis, often resulting in underdiagnosis or delayed diagnosis attributed to insufficient clinical suspicion and diagnostic difficulties [3]. Therefore, there is a significant delay in diagnosing PCD or providing appropriate treatments. Diagnosis of PCD relies on a combination of diagnostic tools including genetic analysis, measurement of nasal nitric oxide (NO), transmission electron microscopy, high-speed videomicroscopy analysis after cell culture, and immunofluorescence [2,4]. The American Thoracic Society and European Respiratory Society guidelines agree that a positive genetic analysis or identification of ultrastructural defects by transmission electron microscopy confirms the diagnosis of PCD [5,6]. Ongoing research and international patient registries aim to improve understanding and treatment options for PCD [2].

Digital high-speed videomicroscopy (DHSV) is a sensitive and specific tool for assessing ciliary function in PCD [7,8,9,10,11], examining ciliary beating frequency (CBF) and ciliary beating pattern (CBP) [12,13]. In young patients suspected of having PCD, frequent ENT procedures necessitate general anesthesia. Despite the need for ciliary samples to confirm PCD diagnosis, concerns persist regarding the potential impact of general anesthesia on ciliary function assessment. Currently, ciliary samples for diagnostic purposes are not obtained under general anesthesia due to the perceived risk of anesthesia-induced alterations in ciliary beat frequency potentially leading to inaccurate diagnoses [14,15].

Currently, there are no studies regarding the assessment of ciliary function by DHSV when sampling is performed under general anesthesia. Our study addressed this critical gap in clinical practice by investigating whether general anesthesia truly influences ciliary function analysis using DHSV. Answering this question is essential for optimizing diagnostic protocols in pediatric patients, potentially allowing for ciliary sample collection during routine interventions under general anesthesia. We sought to provide empirical evidence from real clinical settings to guide future diagnostic practices in suspected PCD cases. Proving that general anesthesia does not affect ciliary function could streamline diagnostics and improve the clinical experience of young ENT patients. Conversely, if anesthesia does alter ciliary function, clinicians should avoid using it to collect samples to avoid inaccurate results. Our aim was to compare the performance of DHSV to analyze ciliary function with and without general anesthesia for sample collection in a small case series. The following three cases present a rare analysis of ciliary function in pediatric patients, both under and outside of general anesthesia. Ciliary function over time was characterized by CBF and CBP using DHSV as co-primary endpoints.

## 2. Case Presentation

### 2.1. Material and Methods

Three pediatric patients referred to the PCD diagnosis center and listed for adenotonsillectomy, adenoidectomy, or tympanostomy tube placement under general anesthesia were included in this case series. Middle turbinate brushing for DHSV analysis was first performed before the induction of general anesthesia as a control sample. Controlateral middle turbinate brushing was then performed under general anesthesia.

- Anesthesia was induced by the inhalation of sevoflurane at an inspired concentration of 6% in a 50% air/oxygen mixture. A bolus of 1–2 mg/kg of propofol and 0.1–0.2 mcg/kg sufentanil was then administered after intravenous access was secured and before endotracheal intubation. After controlling the airway, anesthesia was maintained with sevoflurane at an end-tidal concentration equivalent to 1–1.2 minimal alveolar concentration corrected for age in an air-oxygen mixture. The inspired oxygen fraction was adjusted to maintain the oxygen saturation of the arterial blood above 95%. In addition, throughout the intervention, mean arterial pressure (MAP) was carefully monitored as a reliable indicator of cerebral perfusion. Importantly, MAP was consistently maintained above 33 mmHg to ensure safety and adequate cerebral perfusion. The samples were taken 10 min after airway control so that the concentration of sevoflurane had reached an equilibrium between the different compartments.
- Nasal brushing samples were placed in 2 mL of medium 199 (Thermo Fisher, Waltham, MA, USA) containing an antibiotic solution (1% penicillin/streptomycin (Thermo Fisher, Waltham, MA, USA)) and an antifungal, 1% amphotericin B (Thermo Fisher, Waltham, MA, USA). Ciliary function was assessed immediately (T0) as well as at 1 h (T1) and 3 h (T3) following brushing to study the evolution of a possible anesthetic washout.

Video sequences of ciliated beat edges were recorded using an inverted microscope with a 100× oil immersion interference contrast objective (Axio Vert.A1, Zeiss, Oberkochen, Germany) and a video camera at high speed (CrashCam Mini 1510, IDT Innovation in motion, Pasadena, CA, USA), at a frame rate of 500 hertz (Hz) at a controlled temperature of 37 °C. To record video sequences of cilia beating, 60 µL of the respiratory ciliated edges in medium 199 were placed under the microscope and heated to 37 °C using a heated box (Ibidi, Gräfelfing, Germany) and a microscope lens heater (Tokai Hit, Fujinomiya, Japan), and the temperature was strictly controlled and stable at 37 °C [16].

Only the edges considered normal or having minor projections, measuring at least 50 µm in length, were documented and utilized for ciliary functional analysis (CFA) [17]. Among these edges, only cilia free of mucus and displaying sideways beating profiles were examined. CFA was assessed based on a minimum of three high-quality edges meeting the specified criteria for each condition. For the manual assessment of CBF, cilia, or groups of cilia beating in the sideways profile were identified. The process included counting the number of frames needed to complete five beat cycles, which was then converted to CBF through a simple calculation [12]. A maximum of 10 CBF measurements were obtained from each ciliated beating edge. Ciliated edges that did not allow at least four CBF measurements to be performed were excluded from the analysis. If immobile cilia were present, a CBF of 0 Hz was recorded [18]. The average CBF for each sample was calculated for each treatment condition.

The specific movement of a cilium or group of cilia throughout a complete beating cycle was compared to the normal CBP observed with DHSV [1,2]. Each cilia or ciliary group evaluated was classified as having a distinct normal or abnormal CBP. The percentage of normal CBP in the sample was calculated for each condition [18]. This study received approval from the ethics committee of the University Hospital of Liège 2021-393. Informed written consent was obtained from all patients and their guardians prior to their involvement.

Transmission electron microscopy (TEM) was used to investigate ciliary ultrastructural defects. For TEM analysis, the ciliated cells were initially fixed with 2.5% glutaraldehyde in Sorensen’s phosphate buffer (pH 7.4), then treated with 1.3% osmium tetroxide, dehydrated in graded ethanol, and dried using hexamethyldisilazane. Samples were embedded overnight in a 1:1 1,2-epoxypropan-epon mixture at 4 °C. After polymerization, sections were placed on copper grids and stained with Reynold’s lead citrate.

In addition to TEM, molecular genetic testing was performed to screen for mutations associated with primary ciliary dyskinesia (PCD). PCD is a genetically heterogeneous disorder, typically inherited in an autosomal recessive manner, except for mutations in FOXJ1 (autosomal dominant) and PIHID3, OFD1, and RPGR (X-linked recessive). To date, mutations in more than 50 genes have been implicated in PCD, with the most common being DNAH5, DNAH11, DNAI1, CCDC39, and CCDC40. In this study, a custom gene panel (Gent) was used to screen 125 genes associated with isolated or syndromic congenital heart disease, heterotaxy, and both motile and non-motile ciliopathies.

### 2.2. Case Series

Case #1 was a 7-year-old North African female listed for adenotonsillectomy and tympanostomy tube placement for recurring upper airway obstruction and chronic otitis media with effusion. The patient had a past medical history of congenital heart disease, characterized by a large atrial septal defect and a large interventricular septal defect. These cardiac anomalies were surgically corrected at the age of 3 months. Despite thorough genetic testing, no underlying genetic etiology for the congenital heart defects was identified. She had a previous ENT history of adenoidectomy and tympanostomy tube placement at the age of 2 years. Although she initially experienced relief, symptoms of snoring, mouth breathing, and chronic otitis media with effusion recurred 4 years later, accompanied by auditory impairment.

The patient was not exposed to passive smoking. Extensive diagnostic workup including a sweat test was conducted to rule out cystic fibrosis, which returned negative. Additionally, immunological and allergic assessments were unremarkable. The patient did not respond to conservative treatments including intensive saline nasal irrigation, intranasal glucocorticosteroids, and myofunctional therapy. The ENT examination revealed nasal congestion, chronic otitis media with effusion, and hypertrophy of both the adenoids and tonsils. Tympanometry results were type B curves. Pure tone audiometry showed an average hearing threshold of 25 dB in the right ear and 30 dB in the left ear, indicating mild hearing loss. The PICADAR score was not applicable due to the absence of a persistent wet cough. Given the persistence of symptoms, the patient was listed for adenotonsillectomy and tympanostomy tube placement, with concurrent ciliary sampling, in our ENT department.

The postoperative recovery was uneventful. The patient experienced significant clinical improvement, with the restoration of normal nasal breathing, resolution of snoring, and normalization of hearing. She remains symptom-free with a follow-up period of 5 months.

Case# 2 was a Caucasian male aged 1 year and 5 months listed for adenoidectomy and tympanostomy tube placement for adenoid hypertrophy and chronic otitis media with effusion. The patient had a history of recurrent respiratory issues including multiple episodes of bronchitis, recurrent acute otitis media, and asthma.

There was no history of exposure to passive smoking. Comprehensive immunological and allergic evaluations were unremarkable, ruling out underlying immunodeficiencies or allergic conditions. The ENT evaluation demonstrated bilateral glue ear, adenoid hypertrophy, and nasal congestion. Tympanometry indicated type B curves. Pediatric visual reinforcement audiometry, conducted using sound field speakers, revealed an average hearing threshold of 45 dB. Due to the lack of a chronic wet cough, the PICADAR score could not be assessed. Prior to surgery, the patient received respiratory physiotherapy, selective ß2-adrenergic receptor agonists for asthma management, saline nasal douching, and intranasal glucocorticosteroids. Despite these treatments, the symptoms persisted, and the patient was listed for adenoidectomy and tympanostomy tube placement, with concurrent ciliary sampling. Due to his asthma, he was kept under observation overnight following the surgery to ensure respiratory stability.

The postoperative recovery was uncomplicated. The patient showed significant improvement, with a marked reduction in the frequency of respiratory and ear infections. Hearing returned to normal up to 11 months post-surgery.

Case #3 was an African girl aged 1 year and 3 months listed for adenoidectomy and tympanostomy tube placement for adenoid hypertrophy and chronic otitis media with effusion. The patient had a medical history of situs inversus totalis, a rare congenital condition in which the major visceral organs are positioned as mirror images of their typical locations.

The genetic analysis did not identify any homozygous mutations in genes commonly associated with PCD and situs inversus including *DNAH5*, DNAI1, CCDC39, CCDC40, DNAH11, LRRC6, RPGR, RSPH4A, RSPH9, CCNO, DNAAF1, and *HYDIN*. Nose congestion and ear infections started at 2 months of age. Additional workup successfully ruled out other cardiac diseases, cystic fibrosis, allergies, and immunodeficiencies. Initial treatment included saline nasal irrigation and intranasal glucocorticosteroids. However, these conservative measures did not resolve the patient’s symptoms. The ENT examination showed signs of mouth breathing, chronic otitis media with effusion, and hypertrophy of both the tonsils and adenoids. Tympanometry yielded type B results. Pediatric visual reinforcement audiometry using sound field speakers demonstrated an average hearing threshold of 30 dB, with a notable elevation to 50 dB at 500 Hz. The PICADAR score was deemed inapplicable as the patient did not exhibit a chronic cough. The patient was listed for adenoidectomy and tympanostomy tube placement, with concurrent ciliary sampling. Postoperatively, the patient experienced an improvement in nasal breathing. However, she presented recurrent episodes of otorrhea, particularly on the right side, necessitating ongoing local care. The hearing is currently normal, with a follow-up period of 8 months. Although the patient exhibits organ transposition, PCD diagnosis remains uncertain. Genetic testing including a panel of 125 PCD-related genes and electron microscopy failed to identify any definitive mutations or structural abnormalities typically seen in PCD.

All patients underwent a complete evaluation for suspected PCD including nasal NO measures (only for case #1), genetic analysis (using the PCD heterotaxy gene panel, which covers 125 genes linked to PCD; see Supplementary File S1), electron microscopy analysis, and samples for DHSV taken in the outpatient clinic. Genetic analysis of the PCD heterotaxy panel did not detect any pathogenic mutations, and electron microscopy revealed no structural abnormalities in any of the cases.

In the three cases, no clear underlying cause was identified for the recurrent respiratory infections, aside from adenoid and/or tonsil hypertrophy in Case 1 and Case 2. The diagnosis of PCD in the third case remains uncertain, and she is currently being managed with respiratory physiotherapy and saline nasal irrigation.

### 2.3. Ciliary Beat Frequency

Analysis of CBF in brushing when exposed to general anesthesia (T0, T1, and T3), compared with control pre-anesthesia (control), revealed notable variations compared to the control side (Table 1). In patient #1, there was a consistent increase in CBF under general anesthesia at all time points. Conversely, patient #2 showed a mixed response. While there was an initial increase in CBF observed at 1 h under general anesthesia, a subsequent decrease was noted at 3 h compared to the control side. Patient #3 demonstrated a relatively stable response under general anesthesia. There was a slight increase in CBF at T1 followed by stable CBF levels at T3 compared to the control condition (SA). The CBF for healthy pediatric volunteers in our laboratory was 15.74 Hz ± 1.64 Hertz, measured in a sideway view and pre-culture conditions.

### 2.4. Ciliary Beat Pattern

Analysis of the pattern of ciliary beats as a percentage of normal beats after exposure to general anesthesia at different times (T0, T1, T3) compared to the control side revealed variable effects depending on the patients (Table 2). Patient #1 demonstrated relatively stable ciliary function under general anesthesia, with a non-significant decrease in normal CBP observed at 3 h compared to the control side. Conversely, patient #2 showed a notable decrease over time in normal CBP under general anesthesia compared to saline exposure. Patient #3 showed a relative stable ciliary beat pattern over time with minor variations. The CBP for healthy pediatric volunteers in our laboratory was 86.89% ± 6.83 Hertz, measured in a sideway view and pre-culture conditions.

## 3. Discussion

**Figure 1:**
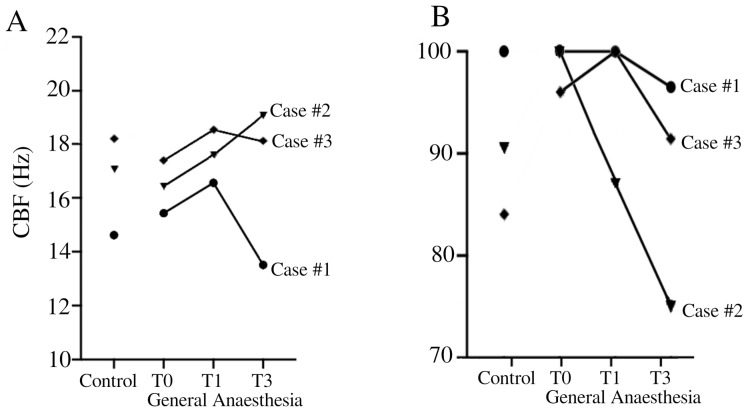
Temporal evolution of CBF (**A**) and CBP (**B**) in Case #1 (●), Case #2 (▼), and Case #3 (♦). CBF (Hz) = ciliary beat frequency in Hertz. CBP = ciliary beat pattern.

Our findings highlighted the variability of respiratory ciliary function under general anesthesia among individuals. Our results emphasize the need for caution when interpreting ciliary function data obtained during general anesthesia. General anesthesia is frequently used in the pediatric population with recurrent respiratory infections. However, taking a sample of ciliary epithelium for ciliary function analysis under general anesthesia is not advisable, despite the temptation to improve patient comfort. The impact of general anesthesia on CBF and CBP is essential to understand the dynamics of mucociliary clearance, particularly in pediatric patients undergoing surgery. Patient #2 showed pronounced changes in CBP and CBF under general anesthesia, indicating disturbances in ciliary function (Figure 1). These results question the impact of general anesthesia on ciliary function.

Concerning general anesthesia, in vivo studies in humans have demonstrated a decrease in CBF after exposure to isoflurane, and interestingly, despite this decrease in CBF, isoflurane did not change the CBP or the proportion of immobile cilia [19,20]. Furthermore, investigations in guinea pigs revealed no significant effects of individual anesthetics such as propofol, midazolam, and sufentanil on tracheal CBF [15,21]. However, when administered in combination, propofol and midazolam exhibited a synergistic interaction, slowing CBF [15]. This finding is consistent with their common target of the GABA_A_ receptor site [15]. On the other hand, the non-significant effect of the propofol-sufentanil combination in the absence of midazolam on CBF suggests a unique pharmacodynamic profile. Notably, similar observations were made in human respiratory cilia samples, where a propofol-alfentanil combination was used [22]. Another study on human ciliated nasal epithelium demonstrated the absence of the effect of halothane on CBF on 24 healthy volunteers and over 3 h [23]. While Marusiakova et al. demonstrated that children with adenoid hypertrophy had significantly lower median CBF than healthy controls [24], we were not able to repeat this finding. All of our patients had normal CBP and CBF without anesthesia compared to our normal laboratory values for children (15.74 Hz ± 1.64 for CBF and 86.89% ± 6.83 for normal CBP) (Figure 1). In fact, Case #3 presented a higher value compared to our normal laboratory values for CBF. The elevated CBF values observed in Table 1 can mostly be explained by the effect of general anesthesia. However, it is important to note that Case #3 exhibited a high CBF prior to anesthesia. It is possible that this patient had a baseline CBF that is higher than our laboratory’s standard. Additionally, this patient’s diagnosis remains uncertain, which could further explain this atypical finding. In addition, for Cases #1 and 3, it is hypothesized that general anesthesia at T0 caused an initial slowing of CBF, followed by a return to pre-anesthesia patterns after the washout period, explaining the higher CBF observed at T1 and T2. In contrast, Case 2 exhibited an initial increase in CBF at T0 and T1, with a return to pre-anesthesia levels later on. While these findings suggest that general anesthesia may influence ciliary function, it is difficult to draw definitive conclusions based on only three cases.

Nonetheless, it is important to acknowledge that patients included in this study have a history of recurrent upper respiratory infections, which raises the possibility of secondary ciliary dyskinesia (SCD). SCD, which can be caused by chronic inflammation or infection, may temporarily impair ciliary function and affect the baseline CBF. This factor could influence our findings, particularly the response of ciliary function under general anesthesia. While our study aimed to assess the impact of anesthesia on ciliary movement, it is essential to recognize that pre-existing conditions like SCD could complicate the interpretation of the observed changes in CBF. Future studies with larger sample sizes and detailed analyses of both primary and secondary dyskinesia are needed to better differentiate these effects.

To date, no similar cases have been published showing the evolution across time of ciliary function using CBF and CBP by DHSV in nasal brushing samples obtained from pediatric patients during general anesthesia for ENT surgery. These cases highlight the importance of carefully interpreting ciliary function data obtained in this context, as anesthesia may influence ciliary activity. The primary objective of this work was to present specific clinical observations in patients suspected of primary ciliary dyskinesia (PCD) who underwent anesthesia. These case reports, while limited in number, provide important insights that contribute to the understanding of this rare condition.

In conclusion, this study reports observations from three individual cases and therefore cannot provide definitive conclusions on the effects of general anesthesia on ciliary beat frequency (CBF) in patients with or without primary ciliary dyskinesia (PCD). The variability observed in CBF across the cases suggests that general anesthesia may influence ciliary function, but further investigation with a larger cohort is necessary to fully understand this relationship. Until more robust data are available, we recommend caution in interpreting ciliary function from samples taken under anesthesia.

## Figures and Tables

**Table 1:** Temporal evolution of ciliary beat frequency (CBF) after exposure to general anesthesia compared to control side pre-operatively. Results are expressed as the median [P25–P75].

CBF, Median [P25–P75]	Control	General Anesthesia		
		T0	T1	T3
Case #1	17.1 [16.0–17.7]	16.5 [14.5–17.7]	17.6 [16.7–18.9]	19.1 [17.7–20.0]
Case #2	14.6 [12.7–16.8]	15.4 [14.1–17.0]	16.6 [14.4–17.1]	13.5 [11.7–13.8]
Case #3	18.3 [15.1–19.8]	17.2 [14.9–18.5]	18.7 [17.2–19.0]	18.1 [14.2–18.5]

**Table 2:** Temporal evolution of ciliary beat pattern (CBP) expressed as a percentage of normal CBP after exposure to general anesthesia compared to saline pre-operatively (control). Results are expressed as a percentage of normal CBP.

CBP, Percentage %	Control	General Anesthesia		
		T0	T1	T3
Case #1	100	100	100	96.5
Case #2	90.5	100	87.0	75.0
Case #3	84.0	96.0	100	91.4

## Data Availability

All data are contained within the article.

## Supplementary Materials

The following supporting information can be downloaded at: https://www.mdpi.com/article/10.3390/diagnostics14212436/s1, File S1: H9.1-OP2-B11: Genpanel Heterotaxie PCD, v2, in voege op 27/06/2022.
