# Supplementary material for: Impact of General Anesthesia on Ciliary Functional Analysis by Digital High-Speed Videomicroscopy in Suspected Primary Ciliary Dyskinesia
Source: Diagnostics (Basel). 2024 Oct 31;14(21):2436. doi: 10.3390/diagnostics14212436 (PMC11544925; doi:10.3390/diagnostics14212436)
Supplement: Supplementary file 1 [file diagnostics-14-02436-s001.zip › diagnostics-3156789-supplementary.pdf]

Heterotaxie PCD (Primaire ciliaire dyskinesie) panel

**versie**	v2 (125 genen)	Centrum voor Medische Genetica Gent

Gene	OMIM gene ID	Associated phenotype, OMIM phenotype ID, phenotype mapping key and inheritance pattern
ACTC1	102540	Left ventricular noncompaction 4, 613424 (3), Autosomal dominant; Cardiomyopathy, hypertrophic, 11, 612098 (3), Autosomal dominant; Atrial septal defect 5, 612794 (3), Autosomal dominant; Cardiomyopathy, dilated, 1R, 613424 (3), Autosomal dominant
ACVR2B	602730	Heterotaxy, visceral, 4, autosomal, 613751 (3)
AK7	615364	?Spermatogenic failure 27, 617965 (3), Autosomal recessive
ALMS1	606844	Alstrom syndrome, 203800 (3), Autosomal recessive
ANKS6	615370	Nephronophthisis 16, 615382 (3), Autosomal recessive
BBS1	209901	Bardet-Biedl syndrome 1, 209900 (3), Autosomal recessive, Digenic recessive
BBS10	610148	Bardet-Biedl syndrome 10, 615987 (3), Autosomal recessive
BBS2	606151	Retinitis pigmentosa 74, 616562 (3), Autosomal recessive; Bardet-Biedl syndrome 2, 615981 (3), Autosomal recessive
BCL9L	609004	No OMIM phenotype
BCOR	300485	Microphthalmia, syndromic 2, 300166 (3), X-linked dominant
BRAF	164757	Melanoma, malignant, somatic, 155600 (3); LEOPARD syndrome 3, 613707 (3), Autosomal dominant; Cardiofaciocutaneous syndrome, 115150 (3), Autosomal dominant; Adenocarcinoma of lung, somatic, 211980 (3); Noonan syndrome 7, 613706 (3), Autosomal dominant; Colorectal cancer, somatic, 114500 (3); Nonsmall cell lung cancer, somatic, 211980 (3)
CBL	165360	Noonan syndrome-like disorder with or without juvenile myelomonocytic leukemia, 613563 (3), Autosomal dominant; ?Juvenile myelomonocytic leukemia, 607785 (3), Somatic mutation, Autosomal dominant
CCDC103	614677	Ciliary dyskinesia, primary, 17, 614679 (3), Autosomal recessive
CCDC39	613798	Ciliary dyskinesia, primary, 14, 613807 (3), Autosomal recessive
CCDC40	613799	Ciliary dyskinesia, primary, 15, 613808 (3), Autosomal recessive
CCDC65	611088	Ciliary dyskinesia, primary, 27, 615504 (3), Autosomal recessive
CCNO	607752	Ciliary dyskinesia, primary, 29, 615872 (3), Autosomal recessive
CENPF	600236	Stromme syndrome, 243605 (3), Autosomal recessive
CEP164	614848	Nephronophthisis 15, 614845 (3), Autosomal recessive
CEP290	610142	Leber congenital amaurosis 10, 611755 (3); Joubert syndrome 5, 610188 (3), Autosomal recessive; Senior-Loken syndrome 6, 610189 (3), Autosomal recessive; ?Bardet-Biedl syndrome 14, 615991 (3), Autosomal recessive; Meckel syndrome 4, 611134 (3), Autosomal recessive
CFAP298	615494	Ciliary dyskinesia, primary, 26, 615500 (3), Autosomal recessive

CFAP300	618058	Ciliary dyskinesia, primary, 38, 618063 (3), Autosomal recessive
CFAP52	609804	Heterotaxy, visceral, 10, autosomal, with male infertility, 619607 (3), Autosomal recessive
CFAP53	614759	Heterotaxy, visceral, 6, autosomal recessive, 614779 (3), Autosomal recessive
CFC1	605194	Heterotaxy, visceral, 2, autosomal, 605376 (3), Autosomal dominant
CFTR	602421	Cystic fibrosis, 219700 (3), Autosomal recessive; Sweat chloride elevation without CF (3); Congenital bilateral absence of vas deferens, 277180 (3), Autosomal recessive; {Pancreatitis, hereditary}, 167800 (3), Autosomal dominant; {Bronchiectasis with or without elevated sweat chloride 1, modifier of}, 211400 (3), Autosomal dominant; {Hypertrypsinemia, neonatal} (3)
CHD7	608892	Hypogonadotropic hypogonadism 5 with or without anosmia, 612370 (3), Autosomal dominant; CHARGE syndrome, 214800 (3), Autosomal dominant
CITED2	602937	Atrial septal defect 8, 614433 (3), Autosomal dominant; Ventricular septal defect 2, 614431 (3), Autosomal dominant
CRELD1	607170	Atrioventricular septal defect, partial, with heterotaxy syndrome, 606217 (3), Autosomal dominant; {Atrioventricular septal defect, susceptibility to, 2}, 606217 (3), Autosomal dominant
DNAAF1	613190	Ciliary dyskinesia, primary, 13, 613193 (3), Autosomal recessive
DNAAF11	614930	Ciliary dyskinesia, primary, 19, 614935 (3), Autosomal recessive
DNAAF2	612517	Ciliary dyskinesia, primary, 10, 612518 (3)
DNAAF3	614566	Ciliary dyskinesia, primary, 2, 606763 (3), Autosomal recessive
DNAAF4	608706	{Dyslexia, susceptibility to, 1}, 127700 (3), Autosomal dominant; Ciliary dyskinesia, primary, 25, 615482 (3), Autosomal recessive
DNAAF5	614864	Ciliary dyskinesia, primary, 18, 614874 (3), Autosomal recessive
DNAAF6	300933	Ciliary dyskinesia, primary, 36, X-linked, 300991 (3), X-linked recessive
DNAH1	603332	Spermatogenic failure 18, 617576 (3), Autosomal recessive; ?Ciliary dyskinesia, primary, 37, 617577 (3), Autosomal recessive
DNAH11	603339	Ciliary dyskinesia, primary, 7, with or without situs inversus, 611884 (3), Autosomal recessive
DNAH5	603335	Ciliary dyskinesia, primary, 3, with or without situs inversus, 608644 (3)
DNAH8	603337	Spermatogenic failure 46, 619095 (3), Autosomal recessive
DNAH9	603330	Ciliary dyskinesia, primary, 40, 618300 (3), Autosomal recessive
DNAI1	604366	Ciliary dyskinesia, primary, 1, with or without situs inversus, 244400 (3), Autosomal recessive
DNAI2	605483	Ciliary dyskinesia, primary, 9, with or without situs inversus, 612444 (3), Autosomal recessive
DNAJB13	610263	Ciliary dyskinesia, primary, 34, 617091 (3), Autosomal recessive
DNAL1	610062	Ciliary dyskinesia, primary, 16, 614017 (3), Autosomal recessive
DRC1	615288	Ciliary dyskinesia, primary, 21, 615294 (3), Autosomal recessive

ELN	130160	Cutis laxa, autosomal dominant, 123700 (3), Autosomal dominant; Supravalvar aortic stenosis, 185500 (3), Autosomal dominant
EVC	604831	Ellis-van Creveld syndrome, 225500 (3), Autosomal recessive; ?Weyers acrofacial dysostosis, 193530 (3), Autosomal dominant
EVC2	607261	Ellis-van Creveld syndrome, 225500 (3), Autosomal recessive; Weyers acrofacial dysostosis, 193530 (3), Autosomal dominant
FOXF1	601089	Alveolar capillary dysplasia with misalignment of pulmonary veins, 265380 (3), Autosomal dominant
FOXH1	603621	No OMIM phenotype
GAS2L2	611398	?Ciliary dyskinesia, primary, 41, 618449 (3), Autosomal recessive
GAS8	605178	Ciliary dyskinesia, primary, 33, 616726 (3), Autosomal recessive
GATA4	600576	Tetralogy of Fallot, 187500 (3), Autosomal dominant; Atrial septal defect 2, 607941 (3), Autosomal dominant; Ventricular septal defect 1, 614429 (3), Autosomal dominant; Atrioventricular septal defect 4, 614430 (3), Autosomal dominant; ?Testicular anomalies with or without congenital heart disease, 615542 (3), Autosomal dominant
GATA6	601656	Atrial septal defect 9, 614475 (3), Autosomal dominant; Persistent truncus arteriosus, 217095 (3); Pancreatic agenesis and congenital heart defects, 600001 (3), Autosomal dominant; Atrioventricular septal defect 5, 614474 (3), Autosomal dominant; Tetralogy of Fallot, 187500 (3), Autosomal dominant
GDF1	602880	Congenital heart defects, multiple types, 6, 613854 (3), Autosomal dominant; Right atrial isomerism (Ivemark), 208530 (3), Autosomal recessive
GJA1	121014	Erythrokeratodermia variabilis et progressiva 3, 617525 (3), Autosomal dominant; Craniometaphyseal dysplasia, autosomal recessive, 218400 (3), Autosomal recessive; Oculodentodigital dysplasia, 164200 (3), Autosomal dominant; Hypoplastic left heart syndrome 1, 241550 (3), Autosomal recessive; Palmoplantar keratoderma with congenital alopecia, 104100 (3), Autosomal dominant; Syndactyly, type III, 186100 (3), Autosomal dominant; Oculodentodigital dysplasia, autosomal recessive, 257850 (3), Autosomal recessive; Atrioventricular septal defect 3, 600309 (3), Autosomal dominant
GPC3	300037	Wilms tumor, somatic, 194070 (3); Simpson-Golabi-Behmel syndrome, type 1, 312870 (3), X-linked recessive
HAND1	602406	No OMIM phenotype
HES7	608059	Spondylocostal dysostosis 4, autosomal recessive, 613686 (3), Autosomal recessive
HYDIN	610812	Ciliary dyskinesia, primary, 5, 608647 (3), Autosomal recessive
INVS	243305	Nephronophthisis 2, infantile, 602088 (3), Autosomal recessive
JAG1	601920	?Deafness, congenital heart defects, and posterior embryotoxon, 617992 (3), Autosomal dominant; Alagille syndrome 1, 118450 (3), Autosomal dominant; Tetralogy of Fallot, 187500 (3), Autosomal dominant

KIF7	611254	Joubert syndrome 12, 200990 (3), Autosomal recessive; Acrocallosal syndrome, 200990 (3), Autosomal recessive; ?Hydrolethalus syndrome 2, 614120 (3), Autosomal recessive; ?Al-Gazali-Bakalinova syndrome, 607131 (3), Autosomal recessive
LEFTY2	601877	No OMIM phenotype
LMNA	150330	Mandibuloacral dysplasia, 248370 (3), Autosomal recessive; Heart-hand syndrome, Slovenian type, 610140 (3), Autosomal dominant; Cardiomyopathy, dilated, 1A, 115200 (3), Autosomal dominant; Restrictive dermopathy, lethal, 275210 (3), Autosomal recessive; Emery-Dreifuss muscular dystrophy 3, autosomal recessive, 616516 (3), Autosomal recessive; Charcot-Marie-Tooth disease, type 2B1, 605588 (3), Autosomal recessive; Emery-Dreifuss muscular dystrophy 2, autosomal dominant, 181350 (3), Autosomal dominant; Hutchinson-Gilford progeria, 176670 (3), Autosomal dominant; Lipodystrophy, familial partial, type 2, 151660 (3), Autosomal dominant; Muscular dystrophy, congenital, 613205 (3), Autosomal dominant; Malouf syndrome, 212112 (3), Autosomal dominant
LRRC56	618227	Ciliary dyskinesia, primary, 39, 618254 (3), Autosomal recessive
LZTFL1	606568	Bardet-Biedl syndrome 17, 615994 (3), Autosomal recessive
MAP2K1	176872	Cardiofaciocutaneous syndrome 3, 615279 (3), Autosomal dominant; Melorheostosis, isolated, somatic mosaic, 155950 (3)
MAP2K2	601263	Cardiofaciocutaneous syndrome 4, 615280 (3), Autosomal dominant
MCIDAS	614086	Ciliary dyskinesia, primary, 42, 618695 (3), Autosomal recessive
MED13L	608771	Impaired intellectual development and distinctive facial features with or without cardiac defects, 616789 (3), Autosomal dominant; Transposition of the great arteries, dextro-looped 1, 608808 (3), Autosomal dominant
MEGF8	604267	Carpenter syndrome 2, 614976 (3), Autosomal recessive
MEIS2	601740	Cleft palate, cardiac defects, and mental retardation, 600987 (3), Autosomal dominant
MKS1	609883	Bardet-Biedl syndrome 13, 615990 (3), Autosomal recessive; Meckel syndrome 1, 249000 (3), Autosomal recessive; Joubert syndrome 28, 617121 (3), Autosomal recessive
MMP21	608416	Heterotaxy, visceral, 7, autosomal, 616749 (3), Autosomal recessive
MRE11	600814	Ataxia-telangiectasia-like disorder 1, 604391 (3), Autosomal recessive
MYH6	160710	{Sick sinus syndrome 3}, 614090 (3); Atrial septal defect 3, 614089 (3); Cardiomyopathy, dilated, 1EE, 613252 (3), Autosomal dominant; Cardiomyopathy, hypertrophic, 14, 613251 (3), Autosomal dominant
NAT10	609221	No OMIM phenotype
NEK8	609799	Renal-hepatic-pancreatic dysplasia 2, 615415 (3), Autosomal recessive; ?Nephronophthisis 9, 613824 (3)

NKX2-5	600584	Hypoplastic left heart syndrome 2, 614435 (3), Autosomal dominant; Tetralogy of Fallot, 187500 (3), Autosomal dominant; Hypothyroidism, congenital nongoitrous, 5, 225250 (3), Autosomal dominant; Conotruncal heart malformations, variable, 217095 (3); Ventricular septal defect 3, 614432 (3), Autosomal dominant; Atrial septal defect 7, with or without AV conduction defects, 108900 (3), Autosomal dominant
NKX2-6	611770	Persistent truncus arteriosus, 217095 (3); Conotruncal heart malformations, 217095 (3)
NME8	607421	Ciliary dyskinesia, primary, 6, 610852 (3), Autosomal recessive
NODAL	601265	Heterotaxy, visceral, 5, 270100 (3), Autosomal dominant
NOTCH1	190198	Adams-Oliver syndrome 5, 616028 (3), Autosomal dominant; Aortic valve disease 1, 109730 (3), Autosomal dominant
NOTCH2	600275	Alagille syndrome 2, 610205 (3), Autosomal dominant; Hajdu-Cheney syndrome, 102500 (3), Autosomal dominant
NPHP3	608002	Nephronophthisis 3, 604387 (3), Autosomal recessive; Renal-hepatic-pancreatic dysplasia 1, 208540 (3), Autosomal recessive; Meckel syndrome 7, 267010 (3), Autosomal recessive
NR2F2	107773	46,XX sex reversal 5, 618901 (3), Autosomal dominant; Congenital heart defects, multiple types, 4, 615779 (3), Autosomal dominant
NSD1	606681	Sotos syndrome 1, 117550 (3), Autosomal dominant
ODAD1	615038	Ciliary dyskinesia, primary, 20, 615067 (3), Autosomal recessive
ODAD2	615408	Ciliary dyskinesia, primary, 23, 615451 (3), Autosomal recessive
ODAD3	615956	Ciliary dyskinesia, primary, 30, 616037 (3), Autosomal recessive
ODAD4	617095	Ciliary dyskinesia, primary, 35, 617092 (3), Autosomal recessive
OFD1	300170	Simpson-Golabi-Behmel syndrome, type 2, 300209 (3), X-linked recessive; ?Retinitis pigmentosa 23, 300424 (3), X-linked recessive; Orofaciodigital syndrome I, 311200 (3), X-linked dominant; Joubert syndrome 10, 300804 (3), X-linked recessive
PITRM1	618211	Spinocerebellar ataxia, autosomal recessive 30, 619405 (3), Autosomal recessive
PKD1L1	609721	Heterotaxy, visceral, 8, autosomal, 617205 (3), Autosomal recessive
PKD2	173910	Polycystic kidney disease 2, 613095 (3), Autosomal dominant
PQBP1	300463	Renpenning syndrome, 309500 (3), X-linked recessive
PRRX1	167420	Agnathia-otocephaly complex, 202650 (3), Autosomal recessive, Autosomal dominant
PTPN11	176876	Noonan syndrome 1, 163950 (3), Autosomal dominant; LEOPARD syndrome 1, 151100 (3), Autosomal dominant; Metachondromatosis, 156250 (3), Autosomal dominant; Leukemia, juvenile myelomonocytic, somatic, 607785 (3)
RAF1	164760	Cardiomyopathy, dilated, 1NN, 615916 (3), Autosomal dominant; Noonan syndrome 5, 611553 (3), Autosomal dominant; LEOPARD syndrome 2, 611554 (3)
RIT1	609591	Noonan syndrome 8, 615355 (3), Autosomal dominant

RPGR	312610	Retinitis pigmentosa, X-linked, and sinorespiratory infections, with or without deafness, 300455 (3); Cone-rod dystrophy, X-linked, 1, 304020 (3), X-linked; Retinitis pigmentosa 3, 300029 (3); Macular degeneration, X-linked atrophic, 300834 (3), X-linked recessive
RSPH1	609314	Ciliary dyskinesia, primary, 24, 615481 (3), Autosomal recessive
RSPH3	615876	Ciliary dyskinesia, primary, 32, 616481 (3), Autosomal recessive
RSPH4A	612647	Ciliary dyskinesia, primary, 11, 612649 (3)
RSPH9	612648	Ciliary dyskinesia, primary, 12, 612650 (3)
SHOC2	602775	Noonan syndrome-like with loose anagen hair 1, 607721 (3), Autosomal dominant
SHROOM3	604570	No OMIM phenotype
SMAD2	601366	Congenital heart defects, multiple types, 8, with or without heterotaxy, 619657 (3), Autosomal dominant
SMAD6	602931	Aortic valve disease 2, 614823 (3), Autosomal dominant; {Radioulnar synostosis, nonsyndromic}, 179300 (3), Autosomal dominant; {Craniosynostosis 7, susceptibility to}, 617439 (3), Autosomal dominant
SOS1	182530	Noonan syndrome 4, 610733 (3), Autosomal dominant; ?Fibromatosis, gingival, 1, 135300 (3), Autosomal dominant
SPAG1	603395	Ciliary dyskinesia, primary, 28, 615505 (3), Autosomal recessive
STK36	607652	?Ciliary dyskinesia, primary, 46, 619436 (3), Autosomal recessive
TBX1	602054	Tetralogy of Fallot, 187500 (3), Autosomal dominant; DiGeorge syndrome, 188400 (3), Autosomal dominant; Conotruncal anomaly face syndrome, 217095 (3); Velocardiofacial syndrome, 192430 (3), Autosomal dominant
TBX5	601620	Holt-Oram syndrome, 142900 (3), Autosomal dominant
TCTN2	613846	Joubert syndrome 24, 616654 (3), Autosomal recessive; ?Meckel syndrome 8, 613885 (3), Autosomal recessive
TTC8	608132	Bardet-Biedl syndrome 8, 615985 (3), Autosomal recessive; ?Retinitis pigmentosa 51, 613464 (3), Autosomal recessive
UBR1	605981	Johanson-Blizzard syndrome, 243800 (3), Autosomal recessive
WDR35	613602	Short-rib thoracic dysplasia 7 with or without polydactyly, 614091 (3), Autosomal recessive; Cranioectodermal dysplasia 2, 613610 (3), Autosomal recessive
ZFPM2	603693	Diaphragmatic hernia 3, 610187 (3); 46XY sex reversal 9, 616067 (3), Autosomal dominant; Tetralogy of Fallot, 187500 (3), Autosomal dominant
ZIC3	300265	Congenital heart defects, nonsyndromic, 1, X-linked, 306955 (3), X-linked recessive; Heterotaxy, visceral, 1, X-linked, 306955 (3), X-linked recessive; VACTERL association, X-linked, 314390 (3), X-linked recessive
ZMPSTE24	606480	Mandibuloacral dysplasia with type B lipodystrophy, 608612 (3), Autosomal recessive; Restrictive dermopathy, lethal, 275210 (3), Autosomal recessive
ZMYND10	607070	Ciliary dyskinesia, primary, 22, 615444 (3), Autosomal recessive

ZNF423	604557	Nephronophthisis 14, 614844 (3), Autosomal recessive, Autosomal dominant; Joubert syndrome 19, 614844 (3), Autosomal recessive, Autosomal dominant

Gene symbols used are according to the HGNC guidelines. For some genes a previously HGNC-approved symbol is in brackets.
Each Phenotype is followed by its MIM number, phenotype mapping key and inheritance pattern.
OMIM release used for OMIM disease identifiers and descriptions: June 06, 2017

Possible phenotype mapping keys
(1) the disorder is placed on the map based on its association with a gene, but the underlying defect is not known
(2) the disorder has been placed on the map by linkage; no mutation has been found
(3) the molecular basis for the disorder is known; a mutation has been found in the gene
(4) a contiguous gene deletion or duplication syndrome, multiple genes are deleted or duplicated causing the phenotype

Brackets, "[ ]", indicate "nondiseases," mainly genetic variations that lead to apparently abnormal laboratory test values (e.g., dysalbuminemic euthyroidal hyperthyroxinemia).
Braces, "{ }", indicate mutations that contribute to susceptibility to multifactorial disorders (e.g., diabetes, asthma) or to susceptibility to infection (e.g., malaria).
A question mark, "?", before the phenotype name indicates that the relationship between the phenotype and gene is provisional. More details about this relationship are provided in the comment field of the map and in the gene and phenotype OMIM entries.
